# Spread pattern of the first dengue epidemic in the city of Salvador, Brazil

**DOI:** 10.1186/1471-2458-8-51

**Published:** 2008-02-07

**Authors:** Florisneide R Barreto, Maria Gloria Teixeira, Maria da Conceição N Costa, Marilia S Carvalho, Mauricio L Barreto

**Affiliations:** 1 Institute of Collective Health, Federal University of Bahia, Salvador, Bahia, Brazil; 2 Oswaldo Cruz Foundation, Rio de Janeiro, Brazil

## Abstract

**Background:**

The explosive epidemics of dengue that have been occurring in various countries have stimulated investigation into new approaches to improve understanding of the problem and to develop new strategies for controlling the disease. The objective of this study was to evaluate the characteristics of diffusion of the first dengue epidemic that occurred in the city of Salvador in 1995.

**Methods:**

The epidemiological charts and records of notified cases of dengue in Salvador in 1995 constituted the source of data. The cases of the disease were georeferenced according to census areas (spatial units) and epidemiological weeks (temporal unit). Kernel density estimation was used to identify the pattern of spatial diffusion using the R-Project computer software program.

**Results:**

Of the 2,006 census areas in the city, 1,400 (70%) registered cases of dengue in 1995 and the spatial distribution of these records revealed that by the end of 1995 practically the entire city had been affected by the virus, with the largest concentration of cases occurring in the western region, composed of census areas with a high population density and predominantly horizontal residences compared to the eastern region of the city, where there is a predominance of vertical residential buildings.

**Conclusion:**

The pattern found in this study shows the characteristics of the classic process of spreading by contagion that is common to most infectious diseases. It was possible to identify the epicenter of the epidemic from which centrifugal waves of the disease emanated. Our results suggest that, if a more agile control instrument existed that would be capable of rapidly reducing the vector population within a few days or of raising the group immunity of the population by means of a vaccine, it would theoretically be possible to adopt control actions around the epicenter of the epidemic and consequently reduce the incidence of the disease in the city. This finding emphasizes the need for further research to improve the technology available for the prevention of this disease.

## Background

The potential severity of dengue and its ability to produce outbreaks in large, complex urban centers makes it a major health issue in almost every continent. Despite the efforts of governments to control new epidemics of this disease, the efficacy of the measures implemented has been very limited [1-3] given that the disease continues to increase in incidence and expand into other areas. Therefore, epidemics and hyperendemicity have become established, with simultaneous circulation of several serotypes of the dengue virus. These situations are the precursors of the occurrence of severe forms of the disease [2].

The outbreak of severe epidemics of hemorrhagic dengue fever in the 1950s directed a large proportion of scientific research towards identifying individual and collective risk factors for the clinical presentation of the disease [4-6] and towards developing clinical and immunological studies aimed at reducing its lethality.

Epidemiological studies on the distribution of dengue in the population were relegated to second place despite the importance of the prevention and control of this disease [7]. This trend resulted in the false premise that the scientific and technological advances in the field of biology, more specifically in the instruments available for the prevention of transmissible diseases (antibiotics, insecticides, vaccines, etc.) would practically extinguish infectious diseases. Nevertheless, the emergence and re-emergence of various infectious diseases, including dengue, and the difficulties found in its control, were proof of the relevance of epidemiological studies and indicated the need to reestablish their value.

The strategies used to combat *Aedes aegypti *that had been previously effective in the combat against urban yellow fever, an arbovirus also transmitted by this mosquito, were found to be ineffective in controlling transmission of the dengue virus in densely populated areas. It should also be taken into account that these measures were transposed into a social and demographic urban situation that was very different from the previous one, without incorporating information on the transformations that had occurred in the epidemiology of the disease. This was particularly so with regard to the spread pattern, which had quite different characteristics from those of either yellow fever or dengue itself in the past.

Among these transformations, the increase in demographic density, the mass production of disposable goods that are discarded into the environment and the greatly increased mobility of populations within and between countries are a few of the most significant, vastly facilitating the circulation of the agents and vectors of transmittable diseases. Although the magnitude of the dengue epidemics of the 1950s in southeast Asia had already provided proof that circulation of this virus had become much more intense than previously observed [2], the basic principles of the technology for vector combat remained unchanged.

The failure of chemical and biological vector control, and the fact that no vaccine against this virus is yet available for use in populations, makes observational epidemiological studies fundamental for acquiring knowledge on the transmission of dengue in the complex urban environments in which the disease currently occurs [8]. Information on the speed of vector transmission and the pathways of virus circulation, as well as identification of the populations and areas most affected, are among the many factors to be evaluated in such studies. Data thus acquired would provide a basis for the application of currently available techniques in the prevention of dengue virus infection. Findings may even point to a need to develop new measures of disease control.

Advances in computerized techniques have allowed improvements to be made in study designs by permitting the implementation and manipulation of large databases, resulting in the acquisition of more detailed information on the behavior of the disease, particularly with respect to the spatial-temporal pathway of disease agents in large cities [9].

In the specific case of dengue, knowledge on the pattern of dissemination may contribute towards directing the implementation of preventive measures that are firmly grounded in epidemiology. Consistent etiological hypotheses may be raised, leading to improved understanding of the reasons for the limited effect that has been observed in the measures currently available for dengue control.

Therefore, the objective of the present study was to identify the characteristics of the spatial-temporal pattern of dissemination in the first dengue epidemic that occurred in a large Brazilian city.

## Methods

This was a spatial-temporal cluster study in which the analysis units consisted of the epidemiological week and census areas [10] in Salvador, capital of the state of Bahia, located in northeastern Brazil. Salvador has a geographical area of 313 km^2 ^and a temperature that ranges from 21 – 37°C (mean 25.3°C). It is situated at latitude 12° 55' 34" south and longitude 38° 31' 12" west. In 1995, the year in which the first dengue epidemic occurred, the city had a population of around 2,300,000 inhabitants, corresponding to a mean density of 7,348 inhabitants per km^2^.

**Figure 1:**
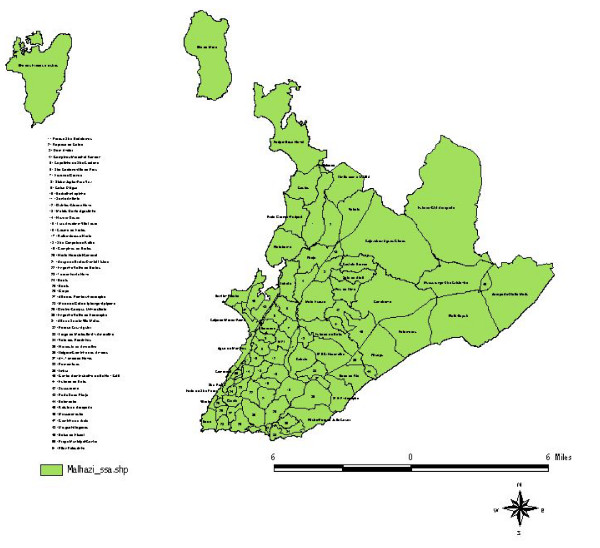
Map of the districts of Salvador (FIBGE-1996).

This city (Figure 1) was founded on the shores of the *Todos os Santos *Bay (All Saints' Bay), and is divided between two topographic levels, the lower city and the upper city. For a long period of its history, the city grew slowly and gradually. However, in the 1960s, this growth rate began to accelerate southwards, specifically in the *Campo Grande *and *Vitória *areas, inhabited by a population of high socioeconomic level; and northwards, where the space was occupied by the lower middle and lower classes.

Today, the historical center of the city, the lower city and neighboring areas are occupied by a low or lower middle class population, and most of the housing is low-rise. With the construction of wide avenues cutting diametrically through the city in valleys, and the population explosion that began in 1960, people from all social classes moved to the northern axis of the municipality and to the Atlantic shoreline. Large shantytowns emerged, taking over spare plots of land in districts occupied by high-rise apartment blocks in which populations of high socioeconomic level resided. Traditional working-class districts such as *Liberdade, São Caetano *and *IAPI*, adjacent to the lower city, also experienced an exponential growth in their populations, with most of the housing in these districts consisting of low-rise buildings or apartment blocks with few floors. This process accelerated further in the last thirty years of the twentieth century, a period when Salvador more than tripled its population, increasing from a population of approximately 700,000 to more than 2.5 million inhabitants [11].

The year 1995 was chosen for this study, because this is the first year for which records of dengue cases in Salvador are available. Therefore, at that time, the entire population was susceptible to the four serotypes of the virus that causes this disease. Since the only serotype isolated in the city in that year and the following year was DEN 2, each individual could only have been affected by dengue once. Furthermore, there were no chemical or biological control measures against *Aedes aegypti *at that time. In other words, the city was naive to infection by the dengue virus and prevention measures, constituting ideal conditions in which to carry out the present study, which, therefore, followed the natural course of a classic dengue epidemic.

In this study, the epidemiological charts and records of notified cases of classic dengue registered at the Bahia State Department of Health (SESAB), originating from both the public and private healthcare networks, were used as sources of data. The criteria adopted by these services throughout Brazil for defining cases of dengue have been standardized by the Ministry of Health according to the recommendations of the Pan American Health Organization, which defines cases of classic dengue as: residents in an area with a confirmed diagnosis of circulating dengue virus, who have an acute febrile disease with a maximum duration of seven days, accompanied by at least two of the following symptoms: headache, retro-orbital eye pain, myalgia, arthralgia, prostration and exanthema [12].

The first cases suspected of being dengue in Salvador occurred in January 1995 and were confirmed using viral isolation (DENV 2 serotype). Next, notification was based on the aforementioned clinical and epidemiological criteria. The investigators considered that the vast majority of these notifications corresponded to cases of classic dengue, bearing in mind that studies carried out in Brazil had found around 80% of serological confirmation among notified cases based on these same diagnostic criteria [13,14].

The data in the fields relating to names, addresses and date of onset of symptoms of the disease were put into a database using the Access software program. Next, duplicated records and those referring to individuals who were not residents of Salvador were excluded, and the spelling of addresses was standardized to avoid any difficulty in georeferencing the cases.

**Figure 2:**
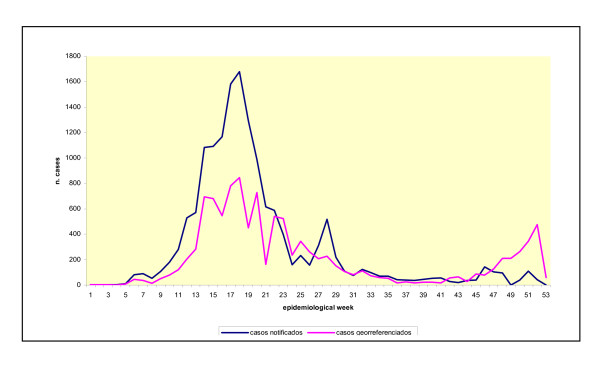
Temporal distribution of the cases of dengue according to epidemiological week. Salvador, Bahia, Brazil, 1995.

Once these procedures had been carried out, this database was integrated with a geocoded base from a geographical data system (Localiza) that had been developed in the Data Technology Laboratory of the Institute of Collective Health. This database contained records of street addresses with the census area codes relating to the 1996 demographic census carried out in Salvador. This system allowed individual addresses to be matched to this code base [15]. With respect to errors in recording addresses in the aforementioned documents, the address was able to be located in 90% of notified cases. The remaining 10% of cases for which this data was missing were possibly random, since following geographic codification, the distribution per epidemiological week remained the same as the distribution of all the registered cases (Figure 2).

The Arcview software program, version 8.1, was used to generate a map showing the distribution of the notified dengue cases per census area in accordance with the following ranges of number of cases: 0, 1–5, 6–16, 17–37, 38–76 and 79–116 cases. Since the figures generated by this program supply only the spatial distribution of the cases, the R-project software program, which contains a statistical module that allows analysis of spatial-temporal patterns, was also used.

Initially, 53 data banks were created, one for each epidemiological week. From these, the cases were mapped sequentially, according to epidemiological week and census area, thereby permitting the pattern of dissemination of the disease to be identified in time and space.

The dissemination pattern of the epidemic was represented graphically using the Kernel density estimation method [16]. Simulations were produced to test bandwidths, considering 500 m, 1 km and 2 km, according to the formula presented below:

$$\hat{\lambda }(s)=\sum_{i-1}^{n}\frac{1}{\tau^{2}}k\left(\frac{\left(s-s_{i}\right)}{\tau }\right)$$

Legend:

l(s) – estimated value per area;

*t *– bandwidth (smoothing factor);

*k *() – kernel weighting function;

s – center of the area;

s_i _– location of the point.

A bandwidth of 2 km was found to be the most adequate to illustrate the progression of the epidemic. A series of 53 Kernel maps were produced, corresponding to each of the epidemiological weeks, with the distribution of the absolute number of notified dengue cases that occurred in each census area during this unit of time. These were used to construct the dissemination pattern of the epidemic. This was also produced in film format, in accordance with the methodology described by Cruz & Carvalho [17].

## Results

Of the 15,458 notifications of dengue recorded by SESAB in the metropolitan region of Salvador in 1995, 14,632 referred to residents of this municipality. In 10,831 cases (74%), georeferencing was possible. In the cases in which georeferencing was impossible, this was because the field corresponding to the address had been left blank or filled in incorrectly.

### Temporal trend

Figure 2 shows the curves referring to the total number of cases recorded and the number of cases that were actually georeferenced in this study. This distribution shows that the greatest concentration of cases occurred in the period between weeks 17 and 20. The apex of the epidemic was at week 18 with 846 and 1,679 records, respectively, on the two curves presented. If only the records that were georeferenced are considered, the number of cases decreased between week 24 and week 42 (the second half of the year), although there was a certain degree of fluctuation in notifications. The maximum during this period was 345 cases (week 25) and the minimum was 16 cases (week 41). Numbers began to increase again in week 47 of the epidemic (mid-November), when 125 cases were registered, and the apex of this new peak (477 cases) was reached in week 52 (end of December). Similar fluctuations were found in the curve for the total number of cases registered in the SESAB system; therefore, the difference in the number of cases did not alter the temporal distribution pattern.

### Spatial distribution

**Figure 3:**
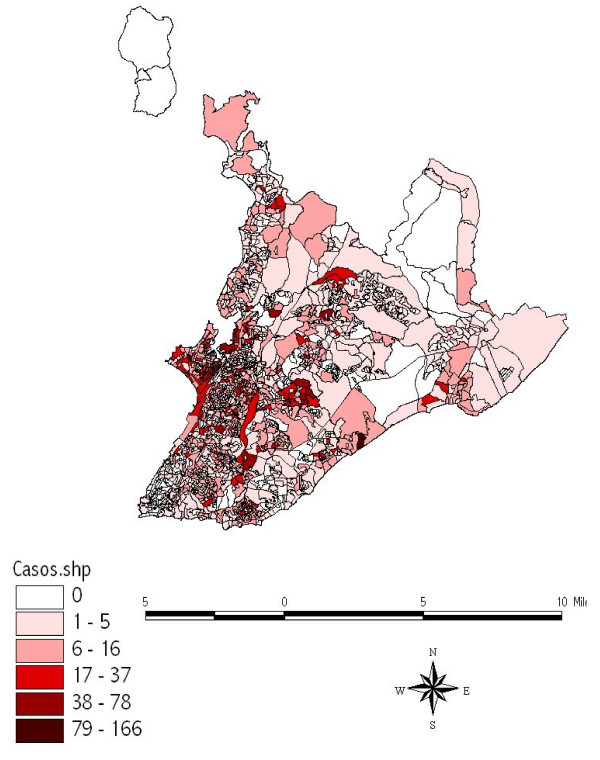
Spatial distribution of notified cases of dengue per census area. Salvador-Bahia, Brazil, 1995.

Cases of dengue were registered in 1,400 (70%) of the 2006 census areas of the city in 1995. The spatial distribution map of these notifications (Figure 3) shows that, by the end of that year, practically the whole city had been affected by this virus, the greatest concentration of cases occurring in the western region of the city where most of the census areas are small because they are densely populated. The map also highlights many geographically large census areas in the region of the lower city in which the incidence of the disease was high, particularly in the *Itapagipe *peninsula, the historical center of the city and other neighboring areas, as well as densely-populated districts in the center of the city such as *Liberdade, São Caetano *and *IAPI*. On the Atlantic coast, there is an area containing many shantytowns that extends from the *Rio Vermelho *section of *Vale das Pedrinhas *to the northeastern part of *Amaralina*, which, albeit small, has similar characteristics of high numbers of cases and dense population. Between these two regions in which the concentration of cases is high, there is another area (*Brotas*), which is at a slightly higher altitude but in which the same high incidence of dengue cases was found. All three of these areas are densely populated and they form a band cutting through the city from west to east, within which most of the census areas registered between 17 and 166 cases (Figures 1 and 3).

In contrast, the southern tip of the city, which is the zone in which the population with the highest socioeconomic power lives and which includes the districts of *Vitória, Graça *(26), *Garcia, Barris *(24), *Barra *and *Ondina *(29), among others, fewer notifications were registered (in the range of 0–5 cases per census area).

Between the districts of the suburban railroad station and the airport and Itapuã, i.e. the entire northern region of Salvador, a pattern was found that was midway between the two types described above, with most census areas reporting between 6 and 16 cases. In addition, there were some census areas in various regions of the city in which no cases were registered. These were generally areas with few inhabitants or none at all such as, for example, Pituaçu Park, the airport/*Stella Maris *area, *Abaeté Park *(*Itapuã*), the City Park (*Pituba*-36) and *Canabrava *(Salvador's former garbage dump), among others (Figures 1 and 3).

### Spatial-temporal distribution

**Figure 4:**
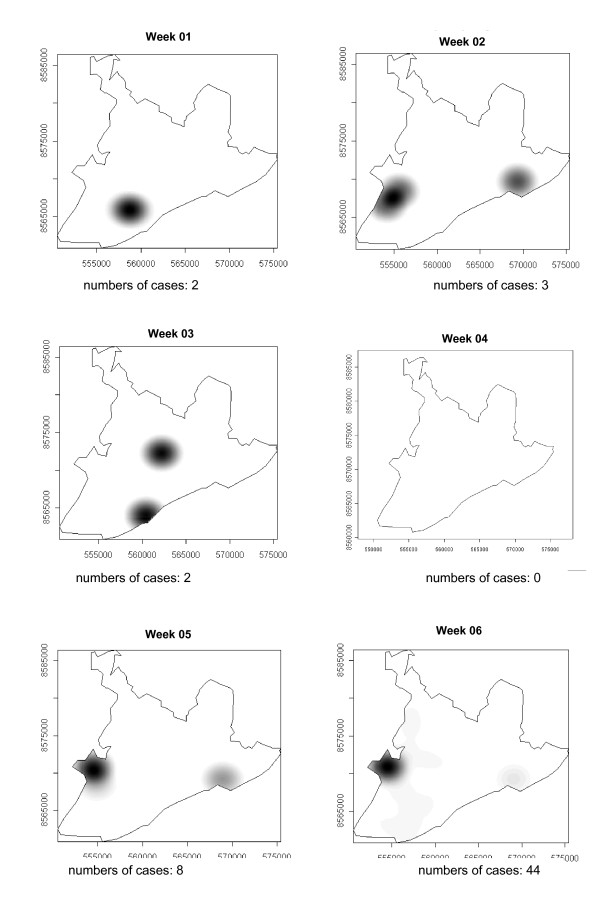
Spread of the dengue epidemic, per epidemiological week. Salvador, Bahia, Brazil, 1995.

**Figure 5:**
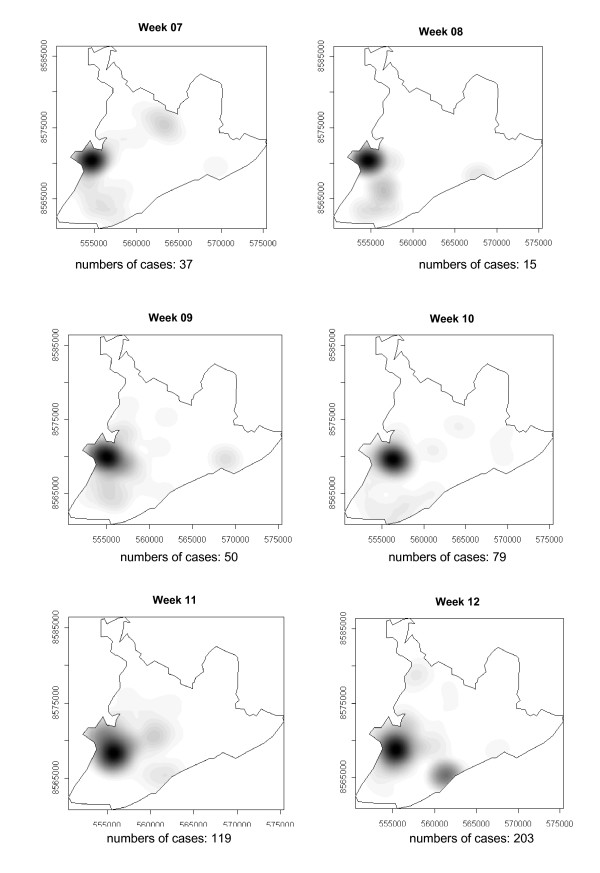
Spread of the dengue epidemic, per epidemiological week. Salvador, Bahia, Brazil, 1995.

**Figure 6:**
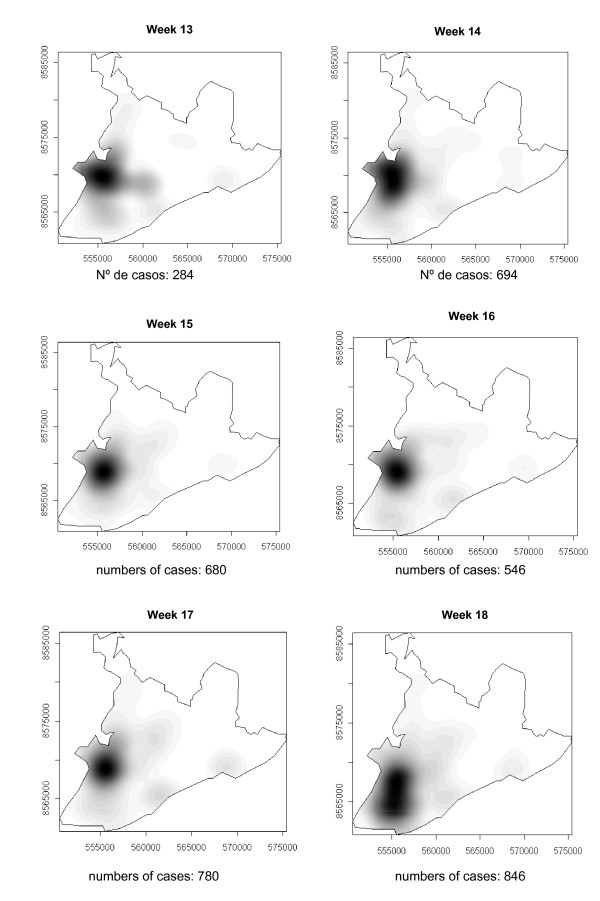
Spread of the dengue epidemic, per epidemiological week. Salvador, Bahia, Brazil, 1995.

**Figure 7:**
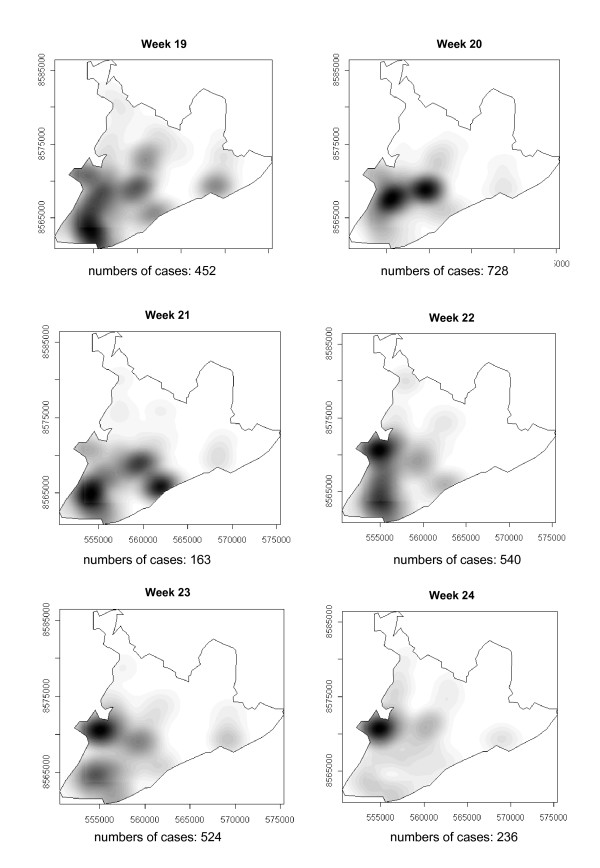
Spread of the dengue epidemic, per epidemiological week. Salvador, Bahia, Brazil, 1995.

**Figure 8:**
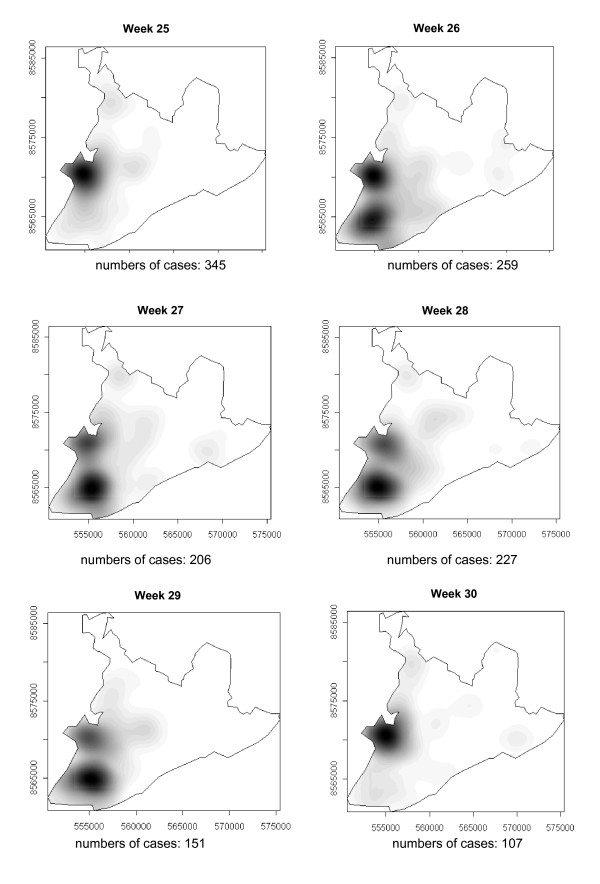
Spread of the dengue epidemic, per epidemiological week. Salvador, Bahia, Brazil, 1995.

**Figure 9:**
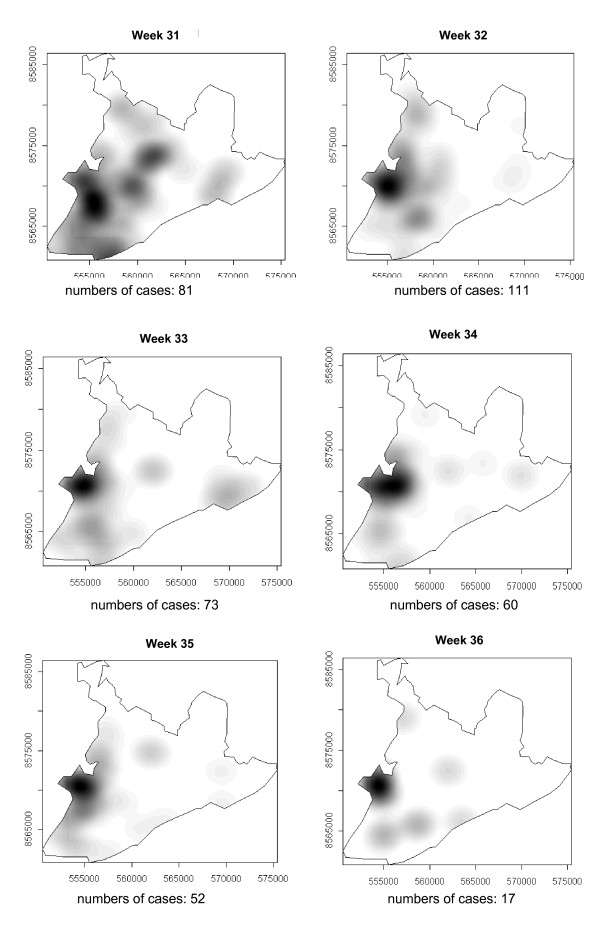
Spread of the dengue epidemic, per epidemiological week. Salvador, Bahia, Brazil, 1995.

**Figure 10:**
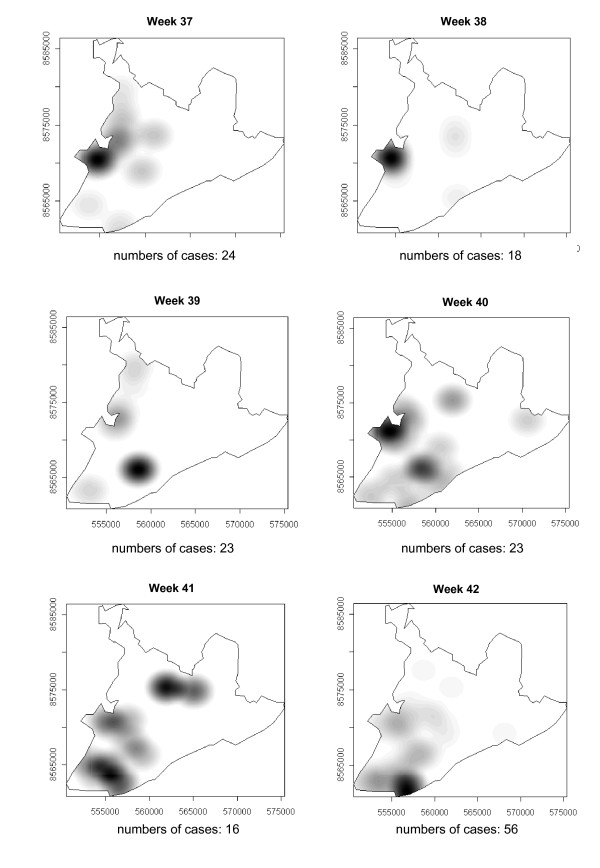
Spread of the dengue epidemic, per epidemiological week. Salvador, Bahia, Brazil, 1995.

**Figure 11:**
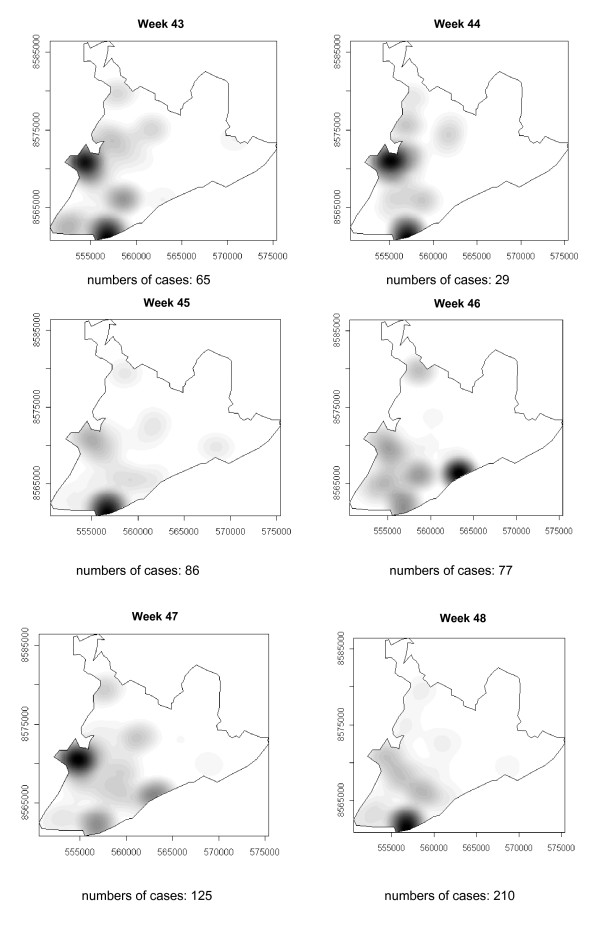
Spread of the dengue epidemic, per epidemiological week. Salvador, Bahia, Brazil, 1995.

The number of dengue cases that occurred in Salvador in 1995 are presented in the consecutive maps shown in Figure 4, 5, 6, 7, 8, 9, 10, 11, 12 according to epidemiological week and location. The first two cases of dengue notified in Salvador occurred in the first epidemiological week of 1995, and these were recorded in two neighboring census areas located in the district of *Pernambués*. During the following two weeks, five cases emerged in four geographically distant districts. After seven days of epidemiological silence, eight cases emerged in week 5, of which five occurred in residents of census areas located on the *Itapagipe *peninsula and other areas of the lower city. This area comprised 44.3% of all notifications up to week 17. In week 18, the epidemic peaked, a spatial shift in the number of cases being clearly seen in that week, although the lower city continued to register cases. From then until week 23, new areas of the city gradually started to be affected, although a concentration of cases continued to be observed in *Itapagipe*. This spread was most intense towards the west (*Baía de Todos os Santos*), the east coast and the southern tip of the city. Nonetheless, some cases were also recorded in other areas of the northern part of the city during this period.

**Figure 12:**
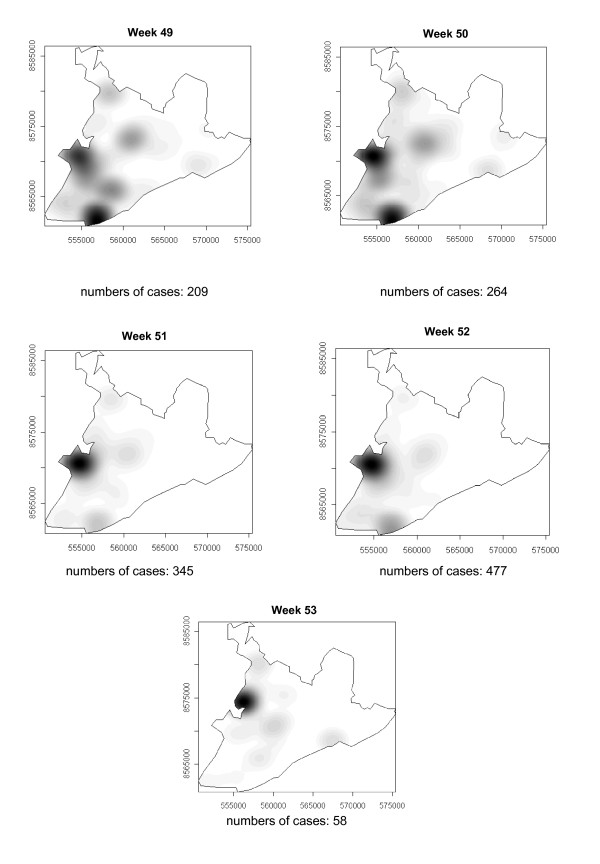
Spread of the dengue epidemic, per epidemiological week. Salvador, Bahia, Brazil, 1995.

From week 24 onwards, the number of notifications began to fall in almost the entire city. However, the greatest numbers of cases continued to be registered in the census areas in the lower city and adjacent areas, being responsible for 541 (39%) of the 1723 notifications occurring between weeks 24 and 32 (the month of August). Among these, around 19% were registered in the *Itapagipe *peninsula. In week 31, there was a great dispersion in notifications, although the number of cases was relatively small (81).

Between weeks 33 and 44, the numbers of cases fluctuated between 16 (week 41) and 73 (week 33), although the trend was downwards. Most of the cases registered during this period also originated from census areas in the lower city, particularly *Itapagipe*, with the sole exception of week 39. In week 38, almost all notifications were registered in this geographical area.

An increase in the number of cases occurred again in epidemiological week 45 and continued until week 52, the total number of registered cases ranging from 77 in week 46 to 477 in week 42. Initially, the concentration of cases was greatest in census areas located on the east coast of the city, although cases were also recorded in *Itapagipe *in every week until the end of the year, indicating that this area had an intense concentration of cases in 5/8 weeks during this period. In week 53, only 58 cases were notified (Figure 4, 5, 6, 7, 8, 9, 10, 11, 12).

**Figure 13:**
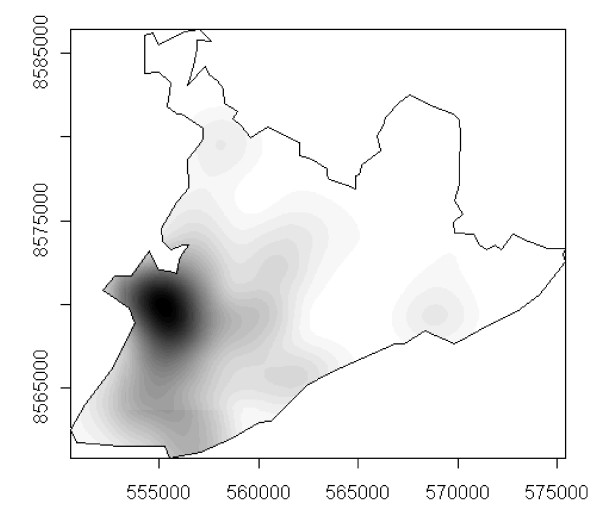
Spatial diffusion patterns of the first dengue epidemic in Salvador, Bahia, Brazil, 1995.

Figure 13, which illustrates the spread pattern of the disease as described in the preceding paragraphs, shows that the process took place in waves originating mainly from the *Itapagipe *peninsula and neighboring areas, all situated in the lower city (from which 35.6% of all cases originated), rapidly irradiating in different directions to other areas of the city.

This dissemination process is more easily visualized in a film produced from this information, which is available as an attachment to this article(see additional file 1).

## Discussion

All studies carried out on data registered in compulsory disease notifications confront difficulties relating to under-notification and diagnostic error when such diagnoses are based only on suspected cases: clinical signs and symptoms and epidemiological connections [18,19]. This is particularly so when the disease in question may be clinically confounded with many others, as is the case with classical dengue. Furthermore, this virus produces non-apparent infections in a high percentage of cases, making it difficult to quantify and follow up the intensity of circulation of the agent without the use of serological techniques with high specificity and sensitivity [20,21]. In spatial studies, these problems are compounded by the variations in the accuracy with which the address field of the notification was filled out, causing many cases to be discarded because of the impossibility of locating the event.

However, in Salvador, this was the first epidemic of dengue, a disease that causes relative panic in view of its potential severity. For this reason, it was quickly accompanied by a major alert and mobilization of the entire population, both through large-scale publicity in the mass media and by communication and information campaigns carried out by the health authorities [22]. This resulted in massive numbers of affected individuals seeking care at the public and private health services. Even considering the hypothesis of a certain percentage of under-registration, under-notification and misdiagnoses, the epidemic scenario, linked to the fact that dengue is a disease for which notification is compulsory, probably contributed to the official notification system being able to record the great majority of cases. Consequently, it is plausible to expect that the space-time trajectory of the apparent cases of the disease in Salvador, as described in this study, may be very close to the actual situation.

It should be emphasized, however, that, although the majority of the studies that use geographical information systems (GIS) also face problems of this type, their results have constituted valuable information for understanding the relationships between the spatial distribution of diseases and environmental and/or sociocultural risk factors [23-26].

Although this study was carried out 10 years after the epidemic in Salvador, the findings are important not only because they reveal some of the principal characteristics of the dynamics of a dengue epidemic in a large urban center of South America where circulation of the dengue virus is relatively recent, but also confirm to what extent a tool such as the GIS may contribute towards improving the surveillance and control of transmissible diseases, particularly if used opportunely. As emphasized by Rotela et al. [26], many countries today are already equipped with remote sensing data, which facilitate the use of the GIS techniques in the routine work of healthcare systems.

Moreover, due to the paucity of information available on the population density of *Aedes aegypti *in the different areas of Salvador, it was not possible to establish a correlation between the building infestation index and the occurrence of cases. Nonetheless, hypotheses coherent with the results found may be raised, based on other information on the environmental sanitation in these areas and on building infestation surveys carried out subsequently.

The clear influence of the *Itapagipe *peninsula on the spread of cases of dengue to almost the entire urban area of Salvador may be seen from the centrifugal waves radiating from that area during almost all of these epidemiological weeks of 1995, and from the high numbers of new cases of the virus, corresponding to more than one-third of all the notifications registered throughout the city. These observations show that the peninsula may be considered the epicenter of the first dengue epidemic in Salvador. As observed in the careful review carried out by Kuno [7], the possibility cannot be discarded of the dengue virus having been introduced in other locations in the city due to intra-urban human mobility and/or the flow of travelers. Nevertheless, the vigorous projection of the cases of dengue over time showed no proof of foci of the disease as productive and long-lasting as those found in the *Itapagipe *peninsula, a finding that strengthens the hypothesis of this district having been the epicenter of the 1995 epidemic.

From the characteristics of the *Itapagipe *peninsula, it is clear that it was not by chance that this area became the center of dissemination of this disease. This region is composed of 13 districts located in the western part of the city, with a population of 145,000 inhabitants mostly of low socioeconomic level, and with poor sanitary conditions and housing. This area includes the district of *Alagados*, which was originally a mangrove swamp and whose name refers to the precarious homes built there on stilts above the floodwaters. The living conditions of its inhabitants, particularly with regard to environmental sanitation, have always been unfavorable, with the constant and abundant presence of mosquitoes, locally referred to as *muriçocas, maruins*, etc., which are a continuous source of complaints from this sector of the population [27].

Although it was not possible to obtain infestation rates for *A. aegypti *because no structured vector control program was available in Salvador during that first year of the epidemic, there have been records of the presence of this mosquito in more than 50% of the city's districts [14]. Although that survey was extremely limited, results showed that, from the beginning of 1995, the dengue virus was already circulating in the city. Nevertheless, the first report containing quantitative information on the infestation levels of this vector dates from 1997, and revealed that, of the 12 sanitary districts of the city, the *Itapagipe *district was the one with the highest building infestation index (23.4%) [28]. These findings indicate that this was the area most likely to become the center of dissemination of the disease.

It is important to note that it was in this area too that the only outbreak of bancroftian filariasis, which was endemic until the beginning of the 1970s, occurred in Salvador. This is a parasitic infection that is also transmitted by a vector (*Culex fatigans*) whose preferred habitat is non-septic cesspits. Although the *Alagados *district has been the target of some sanitation interventions, particularly with regard to the construction of septic tanks, sanitary conditions there remain precarious even today.

Moreover, the homes on the *Itapagipe *peninsula, even those that are not built on stilts, are predominantly low-rise and clustered together, which makes the region densely populated and inhabited by a population of low socioeconomic means. High population density is known to favor transmission of the dengue virus [29,30]. Salvador is a city with a high mean temperature and high humidity, the lower city being one of the areas in which both temperature and humidity are highest. Garbage collection and the supply of water for human consumption continue to be insufficient in *Alagados *[27], thereby providing ideal conditions for the proliferation of many species of mosquitoes, particularly *Aedes aegypti *[31]. These factors must have contributed towards making the district of *Alagados *and the *Itapagipe *peninsula the epicenter of this re-emergent disease and of vector transmission, thereby reproducing a disease pattern similar to those found in the past; however, in a different situation and of different magnitude.

These findings confirm the model developed to explain the occurrence of dengue virus infection in which the inhabited social space is considered to be one of the main factors determining the epidemiological characteristics of the re-emergence of dengue over the past few decades in various countries around the world [6,32-34].

The present findings may be interpreted as contradictory to those reported in a study carried out by Teixeira et al [20] in this same city, which showed no significant differences in risk between areas with favorable or poor living conditions. However, this study was conducted three to four years following the introduction of dengue virus into the city. It is evident that, as the supply of susceptible individuals dwindles in some areas, circulation of the agent will continue to expand relatively rapidly in time and space to other areas, thereby also affecting the higher income districts, although the starting point was the *Alagados *district.

The favorable conditions for dissemination of the disease in the urban space of Salvador continued throughout 1995. Because of a lack of financial resources, the authorities introduced only health education actions [14], which were clearly ineffective in controlling virus circulation, since it only took 120 days (week 18) after the onset of this first dengue epidemic for the virus to be circulating throughout most of the city. Only the more sparsely inhabited areas corresponding to parks, lakes and the airport, etc. remained virus-free.

The centrifugal dissemination of dengue cases in this epidemic characterizes a pattern of diffusion by expansion [35] that was so fast that it resembled the type of expansion that occurs in cases of infectious diseases that are transmitted directly, i.e. spread by contagion.

To the best of our knowledge, no studies have been published in the literature on the diffusion patterns of dengue with which comparisons could be made. Different hypotheses may be put forward, one of them being that the favorable environmental conditions at the epicenter of the epidemic constituted a determining factor, which boosted the intensity of transmission of this virus.

Nonetheless, the magnitude of the epidemics that have been recorded in some of the large urban centers of Brazil and in many other countries in Latin America [2,36,37] is proof that the pattern of re-emergence of the dengue virus is a consequence of the living conditions prevalent in modern cities. In Salvador, the characteristics of the two dengue epidemics that occurred in 1995 and 1996 were no different from those in other Brazilian cities with regard to their magnitude [14]. In the state of Rio de Janeiro, the city of *Nova Iguaçu *has been identified as the entry point for the three serotypes of the dengue virus circulating in Brazil [37] and has always constituted a source of cases. These observations allow the hypothesis to be made that in each urban complex there must be one or more areas that are more receptive to the circulation of the dengue virus, and from which the virus spreads. Therefore, each city may have its own epicenter. This may or may not be the place in which the first cases are detected, considering that in Salvador the first notifications were made in districts at some distance from *Alagados*.

## Conclusion

The questions investigated here merit further evaluation, bearing in mind that this information may have important repercussions in disease surveillance and control. Thus, in large cities, epidemiological surveillance should perhaps not be limited to operating only in the locations in which the first cases arise, but should also consider the productivity of notifications with a view to guiding the vector combat teams.

One fundamental problem for public health, which has been well-established in this and other studies [36,38] concerns the speed and intensity of dengue virus transmission and the limited capacity to reduce the populations of *A. aegypti *to levels at which the disease would be under control using currently available techniques. Even when these actions have been carried out adequately, the complexity of large modern cities with many underprivileged areas, as well as other problems, hinders the success of these programs. Unfortunately, various cycles of combat of this vector are necessary to reduce infestation levels to those incompatible with viral transmission. The Brazilian program of dengue control recommends that each cycle of combat of the *Aedes aegypti *should be of approximately 60 days duration, whereas virus transmission, as may be seen in this study, expands in a few weeks.

Our results suggest that, if there were a more agile control instrument that was capable of rapidly diminishing the vector population, or of increasing group immunity in the population by means of a vaccine, a rapid control action could theoretically be erected around the epicenter of the epidemic, consequently reducing the incidence of the disease in the city. Therefore, in view of the epidemiological importance of dengue in Brazil, it is important for the country to increase its investments in research in order to develop new techniques for the control of this disease.

## Competing interests

The author(s) declare that they have no competing interests.

## Authors' contributions

FRB, MGT e MLB conceived the study and contributed to the analysis and data interpretation. FRB was responsible for data collection and wrote the first draft of the manuscript. MSC contributed to the spatial analysis and MCNC with the data analysis and the write us of the manuscript. All authors reviewed and approved the final version of the manuscript.

## Pre-publication history

The pre-publication history for this paper can be accessed here:



## Supplementary Material

Additional File 1 **Spatial diffusion patterns of the first dengue epidemic in Salvador, Bahia, Brazil, 1995**. A film produced from this information, which is available as an attachment to this article. View in windows media player. Click here for file
